# Review of optical reporters of radiation effects *in vivo*: tools to quantify improvements in radiation delivery technique

**DOI:** 10.1117/1.JBO.28.8.080901

**Published:** 2023-08-08

**Authors:** Jacob Sunnerberg, William S. Thomas, Arthur Petusseau, Matthew S. Reed, P. Jack Hoopes, Brian W. Pogue

**Affiliations:** a Dartmouth College, Thayer School of Engineering, Hanover, New Hampshire, United States; b University of Wisconsin–Madison, Department of Medical Physics, Madison, Wisconsin, United States; c Dartmouth College, Geisel School of Medicine, Hanover, New Hampshire, United States

**Keywords:** radiotherapy, fluorescence, phosphorescence, reactive oxygen species, radiation

## Abstract

**Significance:**

Radiation damage studies are used to optimize radiotherapy treatment techniques. Although biological indicators of damage are the best assays of effect, they are highly variable due to biological heterogeneity. The free radical radiochemistry can be assayed with optical reporters, allowing for high precision titration of techniques.

**Aim:**

We examine the optical reporters of radiochemistry to highlight those with the best potential for translational use *in vivo*, as surrogates for biological damage assays, to inform on mechanisms.

**Approach:**

A survey of the radical chemistry effects from reactive oxygen species (ROS) and oxygen itself was completed to link to DNA or biological damage. Optical reporters of ROS include fluorescent, phosphorescent, and bioluminescent molecules that have a variety of activation pathways, and each was reviewed for its *in vivo* translation potential.

**Results:**

There are molecular reporters of ROS having potential to report within living systems, including derivatives of luminol, 2′7′-dichlorofluorescein diacetate, Amplex Red, and fluorescein. None have unique specificity to singular ROS species. Macromolecular engineered reporters unique to specific ROS are emerging. The ability to directly measure oxygen via reporters, such as Oxyphor and protoporphyrin IX, is an opportunity to quantify the consumption of oxygen during ROS generation, and this translates from *in vitro* to *in vivo* use. Emerging techniques, such as ion particle beams, spatial fractionation, and ultra-high dose rate FLASH radiotherapy, provide the motivation for these studies.

**Conclusions:**

*In vivo* optical reporters of radiochemistry are quantitatively useful for comparing radiotherapy techniques, although their use comes at the cost of the unknown connection to the mechanisms of radiobiological damage. Still their lower measurement uncertainty, compared with biological response assay, makes them an invaluable tool. Linkage to DNA damage and biological damage is needed, and measures such as oxygen consumption serve as useful surrogate measures that translate to *in vivo* use.

## 1 Introduction

**Fig. 1:**
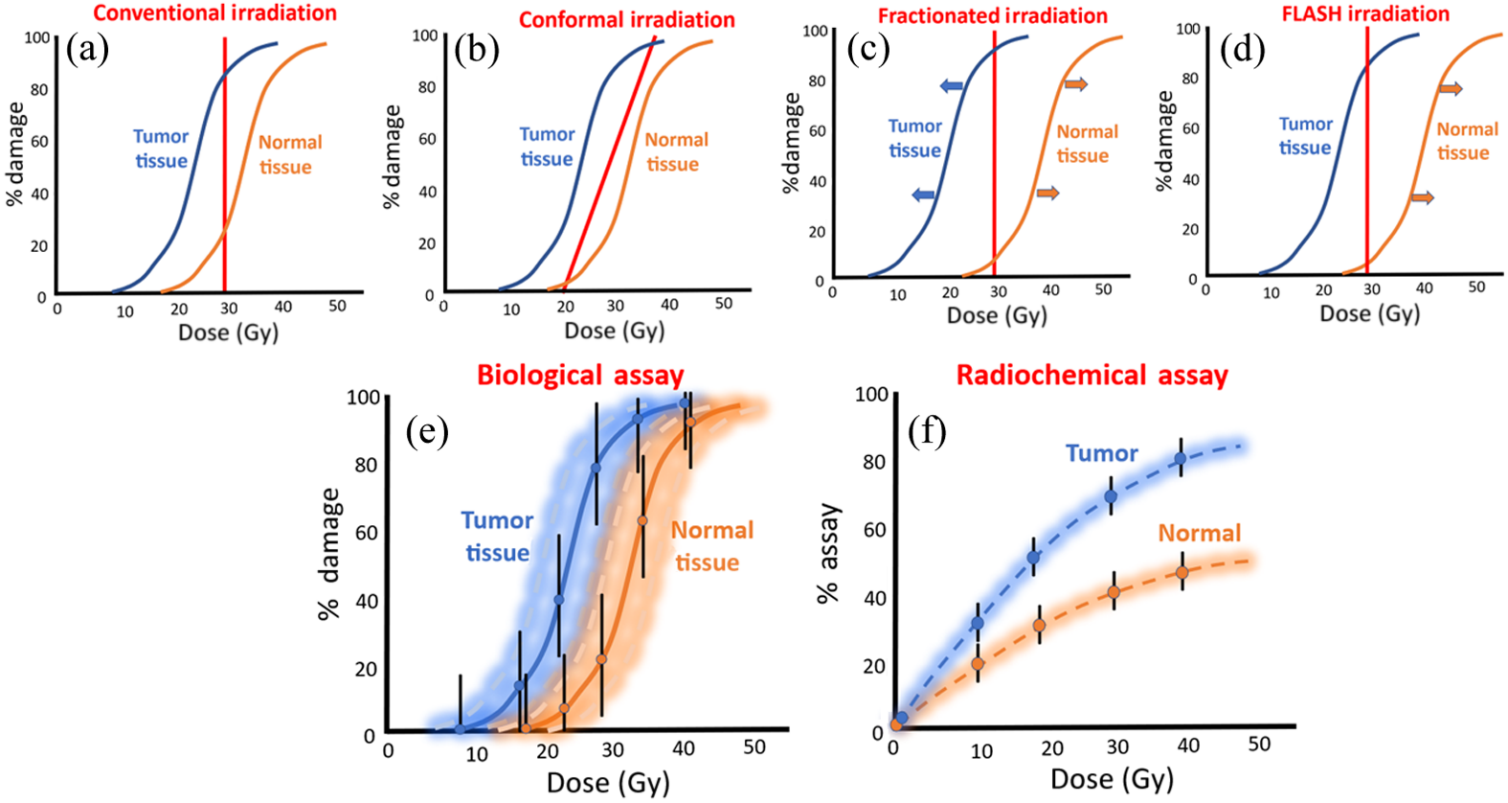
Treatment dose schemes used and being examined and optimized for (a) maximizing the therapeutic ratio of tumor damage to normal tissue damage; (b) with the concept of conformal therapy, maximizing dose to tumor while minimizing it to normal tissue; (c) fractionated therapy, allowing for repair to increase the resistance of normal tissues; and (d) FLASH radiotherapy, providing a protective effect to normal tissues. (e) The problematic variance of biological assays is illustrated conceptually with large error bars and therefore overlapping confidence intervals for tumor and normal tissue assays. (f) The comparatively smaller variation leads to smaller error bars and more statistically separable data to allow for more definitive conclusions when varying irradiation parameters.

Modern radiotherapy is based on the concept of maximizing the therapeutic ratio of damage to tumor tissue relative to normal tissues through (i) the choice of radiation used, (ii) the conformality of delivery, and (iii) the temporal choices in delivery.^1^^–^^3^ Conceptional illustrations of the ways in which this therapeutic ratio might be optimized are illustrated in Fig. 1, with the tumor and normal tissue responses ideally separated at an optimal choice of delivered dose or methodology, allowing for the maximal separation of normal and tumor tissue damage [Fig. 1(a)]. Although many methods of parameter optimization exist within radiotherapy, the ability to optimize the delivery choices requires that an objective assay of damage or tissue health be used. Illustrations of the therapeutic response curves for conformal delivery, fractionated therapy, and FLASH radiotherapy are shown in Figs. 1(b)–1(d), respectively. The ideal and most trusted assay for radiation therapy response in humans is simply survival or surrogates, such as tumor response rate, disease free status, or tumor volume change. However, as new therapy choices are developed and tested in animal models, practical limitations often require that shorter term surrogates be used in a manner that allows for more rapid iterative development of optimized therapy. DNA strand breaks and shorter-term functional assays are commonly applied in experimental studies to assess the acute effects of a modified radiation treatment approach, yet these may be less than ideal because of the high variability in response or in the accuracy of the assay, as conceptually illustrated in Fig. 1(e). It is not uncommon to see biological function assays with a standard deviation in repeated measures >20% to 40% of the mean value, indicating that small changes in therapeutic ratio of 10% to 20% might not be reasonably quantifiable without substantial animal numbers involved, leading to an extremely high cost and labor. Additionally, it is common to have significantly large error bars, and the confidence intervals of tumor and normal tissue response are very wide. This variability in the response measure ultimately limits the accuracy of conclusions from an assay, and any meaningful answers are limited based on the number of animals used. In comparison, surrogate assays can be less quantitatively variable, and they directly sample acute factors such as:

(1) immediate electrons and molecular free radicals produced from hydrolysis
(2) reactive species produced with longer lifetimes
(3) molecules consumed by reactions
(4) biomolecular alterations from these reactions
(5) short-term biological effects.

In this review, some of the key optical reporters for indirect or surrogate measures of radiation damage are assessed, with an eye toward concluding that assays can provide objective accurate information about treatment optimization in FLASH radiotherapy. In particular, a large number of these assay methods use optical reporters or signals, so these are focused on in this review.

**Fig. 2:**
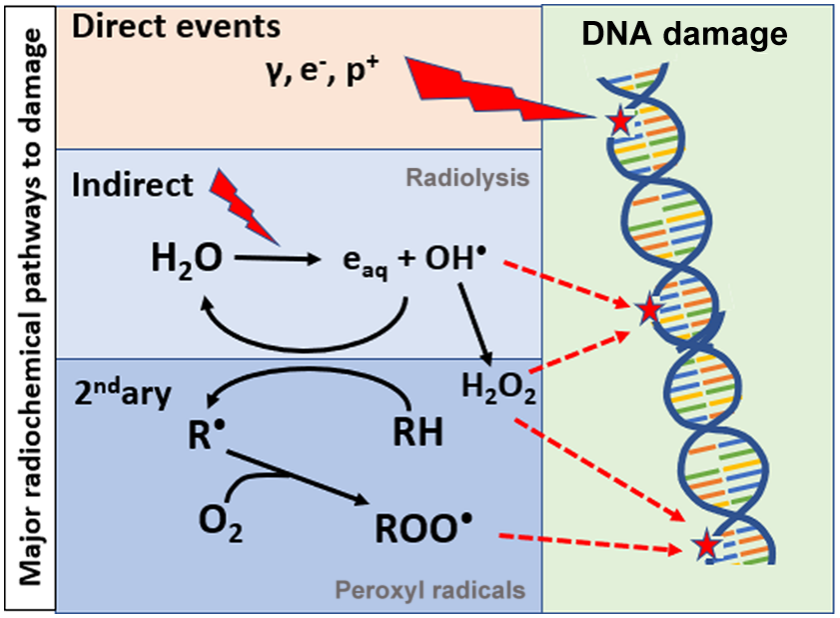
Radiation induced damage is illustrated as direct DNA damage (top) or indirect damage (bottom), each of which is largely proportional to the dose delivered. However, the microenvironment can alter the indirect damage more due to the highly complex interactions with lipids, proteins, and oxygen. The biochemical pathways of radiation-induced hydrolysis and damage are amplified by molecular oxygen, and the peroxyl radicals [ROO•] are a dominant factor in the biological effects.

The most dominant radiation-induced effects that lead to biological damage are outlined in Fig. 2, with the initial radiation dose delivery event being either “direct” DNA damage or “indirect” damage via a more complex cascade of radicals, with the former being the most dominant. Although direct damage is largely thought to dominate radiobiological death via DNA double strand breaks (DSBs), the indirect pathway contributes significantly as well. The exact proportions of each are not well known and likely vary considerably with the tissue and radiation plans. The indirect pathway is dominated by radiolysis of water molecules into hydrogen-based radicals and subsequent hydrogen peroxide ($H_{2}O_{2}$). Reaction with oxygen is possible to create further reactive oxygen species (ROS) and longer-lived reactive molecules, such as $H_{2}O_{2}$. Described here is a simplified model of a very complex set of events, presenting mostly the core pathways that are thought to dominate in radiation biology effects from these radiochemical events. The hydroxyl radical is thought to dominate the reaction with substrate organic molecules, RH, being ionized into R•, which further leads to peroxyl radicals ROO•, from reaction with molecular oxygen. The immediate or downstream effects of these ROS molecules are thought to be genomic, epigenomic protein, and lipid damage in the cells. Each of these transitions has multiple other pathways, but this simplified model presents the dominant core of pathways as identified in numerous studies.^4^^,^^5^ This illustrates what have been postulated as the key inputs or outputs that could be measured, and the structure of this review follows this figure.

**Fig. 3:**
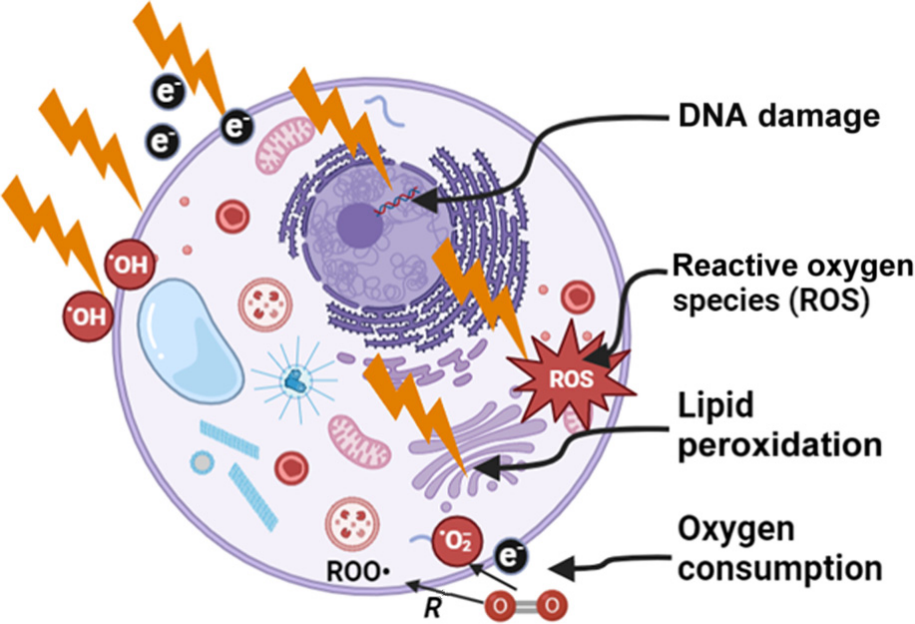
Outline of the major types of optical reporter probes that have been or might be used as assay of radiation effects, including (1) DNA damage, (2) ROS reporter probes, (3) lipid peroxidation probes, and (4) sensors of oxygenation change.

Optical reporters have been a choice for the assessment of radiation damage and effects for as long as radiation studies have been in existence, largely because optical molecular reporters are uniquely resonant with the energy levels of molecular bonds and because of the abundance of visible ways to quantify the assay. In this review, we categorized the choices of damage reporters in terms of those listed in Fig. 3, as reporters of

(1) ROS molecules (i.e., $H_{2}O_{2}$, OH•, and $O_{2}^{-}$)
(2) protein or lipid damage
(3) oxygen consumption
(4) DNA damage
(5) acute biological effects.

These classifications are a practical way to separate out three categories of reporters that, in reality, are not cleanly separated. The unique reporter delineation between each of them is not as clear as desired or as always presented, but these subtypes have a definition that is based around their mechanism and timeline of development. The following series of sections follows the outline of Fig. 3.

*In vitro* assays can rarely completely recapitulate the measure of damage of *in vivo* systems because of the enormous Milieux of organic and inorganic molecules that contribute to the biological responses to radiation and the ultimate damage. However, in many cases, the goal of the assay is simply to assess the magnitude of the proportionate damage increase or decrease, why there is a difference in this between normal and tumor tissues, and how damage changes from different irradiation schemes. It is unlikely that any of these assays would fully reflect biologically driven responses, such as immune involvement, so they are designed or chosen to provide an early timepoint physical–chemical response that is quantifiable with high accuracy. Thus the focus in this paper is on the quantitative proportionate measure of acute damage and how it may be quantified with different techniques of radiation delivery, such as temporally fractionated, ultra-high dose rate (UHDR) FLASH radiotherapy, or spatially fractionated radiotherapy.

## 2 Established DNA Damage Reporters

A comprehensive summary of *in vitro* assays of radiation damage was recently published by Adrian et al.;^6^ it includes most of the biochemical and biological assays of DNA damage that involve antibody assay, such as western blot, enzyme-linked immunosorbent assay, flow cytometry of cells, immunocytochemistry of cell aspirates, and immunohistochemistry staining of slides. The most traditional ways to assay the fundamentals of DNA damage are via comet assay, the more specific DSBs via terminal deoxynucleotidyl transferase-mediated d-UTP nick end labeling (TUNEL) assay, or downstream effects by phosphorylation via γ-H2AX. These are described briefly here because of their seminal role as *in vitro* gold standards of radiation damage, although they are not widely extensible to *in vivo* use.

### 2.1 Comet Assay

Single-cell gel electrophoresis, commonly referred to as the comet assay, is one of the oldest and most established methods of analyzing strand breaks in DNA. Here cells are embedded in agarose on a coated glass slide and lysed with non-ionic detergents and a high salt concentration, which removes cell membranes and disrupts histone protein interactions to leave unobstructed DNA with intact supercoiling.^7^ These slides are placed in an electrophoresis chamber, which pulls the negatively charged DNA toward a positively charged cathode. DNA strand breaks result in relaxed DNA coils that lag behind the nucleoid, resulting in the assay’s characteristic comet-like head and tail that can be stained and viewed with fluorescence microscopy. DNA damage can be characterized by quantifying the tail length as well as the ratio of the tail/head intensity. The assay presents a sensitive method that can be performed in under 24 h, though it also has key limitations, such as the need for single-cell suspensions, large data sets to verify if the damage is homogeneous, and varying methodologies in both performing the assay and analyzing the results. Although the original assay was performed in neutral conditions,^8^ the alkaline assay^9^^,^^10^ became more widely utilized as the high pH further interferes with intramolecular interactions,^11^^,^^12^ allowing alkali-labile sites and single-strand breaks to also result in increased intensity and thus allowing a wider variety of damage to be detected. Several adaptations to this method have been published, such as the use of irradiated blood^13^ or lymphocytes extracted after *in vivo* irradiation to be used for an *ex vivo* comet assay of damage, providing a useful *in vivo*/*ex vivo* assay for DNA damage assessment. Other adaptations have been published that use purified exogenous enzymes highly selective for certain types of damage to digest DNA at those sites, and the resulting increase in tail intensity can be used to quantify particular types of damage.^14^

### 2.2 TUNEL Assay

A further development was a specific assay for DNA DSBs, called the TUNEL assay,^15^ which is attractive for the ease of use, modest cost, and ability to quantify using flow cytometry. The labeling method uses terminal deoxynucleotidyl transferase, a unique immune cell-derived DNA polymerase,^16^ to attach a modified thymine analog with fluorescent tag, known as d-UTP, to the 3′-OH ends of the DSBs. These fluorescent labels can be bound to the nucleotides either directly or indirectly using a chemical label bound to a fluorescent label or antibody. The assay is thought to be specific to DSBs. Since its development in the 1990s, the TUNEL assay has become an established, accurate, and versatile method of DSB detection. Procedure modifications continue to be published, and an extensive set of fluorophores and tags are commercially available. Nucleotide binding to biotin tags is detected using a fluorescent streptavidin conjugate. Although the signal specificity benefits from biotin–streptavidin signal amplification, these assays still require additional treatment to address endogenous biotin and background staining that can be the limiting factor in their sensitivity. However, the major attractions are that Brd-U-based assay reagents have a relatively low cost compared with other TUNEL assay reagents and there is high sensitivity from the comparatively high Brd-U incorporation rate. Still, fixation and permeabilization steps are needed for antibody binding, and they can be reduced by the tissue properties.

### 2.3 Halo Assay

A faster assay of DSBs was needed and stimulated the development of the Halo assay and then the fast halo assay.^17^^,^^18^ This involved a single-cell method in which imaging and counting of the cells were done and an alkaline-halo was induced around the cell. The method was based on observations that broken DNA fragments from cells could be prepared and deproteinized and were radially separated from intact DNA with a simple incubation in a denaturing NaOH solution. Then without the need for electrophoresis, the process was achieved by solvent gradient. Fluorescence microscopy was used to image circular “halos” of highly fragmented DNA surrounding the bright nuclear remnants. The size of the halos is a direct function of the level of DNA breaks.^19^

### 2.4 γ-H2AX Assay

Further improvement in the more subtle quantification of biological response damage came with a measure of a very early biochemical cellular response to DNA DSBs, via a measure of the molecule γ-H2AX. This γ-H2AX is formed on phosphorylation of the histone variant H2AX at the Ser-139 site. To maintain normal cellular function and ensure long-term survival, DSBs are responded to immediately by repair pathways, and γ-H2AX is the product of H2AX phosphorylation, which is a vital step in DNA damage repair because it signals the accumulation of repair proteins and the repositioning of nucleosomes. These form cohesions and act as anchors between DSB ends, keeping them close to one another to preventing false reattachment and/or loss of genetic information.^20^ Methods of quantification of intracellular γ-H2AX foci have been developed to measure damage at the local level during repair processes. Rogakou et al.^21^ found that γ-H2AX levels rise rapidly within a few minutes post-irradiation and peak at 10 to 30 min. In more recent years, *in vivo* and *ex vivo* methods for analyzing γ-H2AX foci have been developed and implemented.^22^^,^^23^ The process of *in vivo* irradiation followed by biopsy and *ex vivo* processing has been used, although the post-processing stage of immunofluorescence can limit the timing and ease of use. However, this is still one of the most acute biological measures of DSB damage in radiation studies today.

### 2.5 Acridine Orange

AO is a dye that has been widely used throughout cellular biology and genetics to assess DNA localization and damage.^24^ As a cell permeating dye that is readily dissolved in an aqueous solution, it binds to double strand DNA and emits green fluorescence to commonly visualize the nuclei of cells (excitation 500  nm/emission 526 nm) and then binds to single-strand DNA or RNA and emits a red fluorescence (excitation 460  nm/emission 650 nm).^24^^,^^25^ Used in radiation damage studies since publications in the 1960s, it concentrates in acidic organelles within the cell, with the uptake dependent on pH, which is often used as a viability dye *in vitro* and is very commonly used with ethidium bromide (EB) as a dual stain for apoptosis death of cells (excitation 518  nm/emission 605 nm). In this assay, live cells have green fluorescence from AO, which is highly cell permeable, whereas dying cells with broken membranes show red fluorescence from EB as it is not highly permeable unless there is damage. However, the conversion of spectrum of AO itself from DNA to RNA binding has been used as a linear assay of radiation dose and to compare the radiobiological efficacy of different types of radiation and repair kinetics.^26^^,^^27^

*In vivo* use of AO has been incidental, although it was used in human studies for early diagnostic studies.^28^ This use predates much of modern toxicity testing, so it has not been widely used or tested in humans as a diagnostic agent in recent years. In an unrelated development though, a series of studies have described the use of AO as a radiation therapy potentiator or sensitizer,^29^^–^^32^ so its safety profile for human use has been tested in a large number of subjects. The function of this is presumably related to its DNA localization ability and its ability to facilitate light energy transfer^33^ or free radical chemistry within the DNA. The use of AO as an *in vivo* reporter of damage has only been shown *in vitro* via individual cell imaging and *in vivo*.^34^ There is also documented use in plants with the translation of the AO/EB assay *in vivo*.^35^ Given that it is already used in humans as a therapeutic, it has potential to be used as an assay in experimental work with even human translation potential.

## 3 ROS Reporters

### 3.1 Small Molecule ROS Reporters

There are many fluorescent reporters of ROS that have been developed and examined over decades. The major complication with their use is that most do not have exact specificity to a particular ROS species, and so the cannot uniquely identify exactly the radiochemistry uniquely. However, the major sensors studied are summarized here.

### 3.2 Fluorescence

Amplex Red and Amplex Ultra Red are commonly applied molecules in ROS fluorescent assays^36^ because these substrates have proven to exhibit the desirable effect of both high specificity and sensitivity of $H_{2}O_{2}$ down to a low near 50 nM. Amplex Red has been used with cell cultures, excised tissue, and some *ex vivo* tests.^37^^,^^38^ Amplex Red/UltraRed are fluorogenic substrates with a very low background fluorescence, and in the presence of horseradish peroxidase, both substrates react 1:1 stoichiometrically with $H_{2}O_{2}$ to produce highly fluorescent resorufin. Standard Amplex Red has a peak excitation/emission of 571/585  nm, whereas Amplex UltraRed has peaks at 568/581  nm and increased fluorescent yield and molecular sensitivity to $H_{2}O_{2}$. As a means of investigating radiation-induced ROS generation, Amplex Red has been primarily used *in vitro* and in cell-free environments. Though limited, Amplex Red/UltraRed have been used *in vivo* in concert with microdialysis to detect $H_{2}O_{2}$ and superoxide in human skeletal muscle^37^ and in rodent models.^38^ This method allows for accurate $H_{2}O_{2}$ concentration measurements localizable to an organ or tissue of interest.

**Fig. 4:**
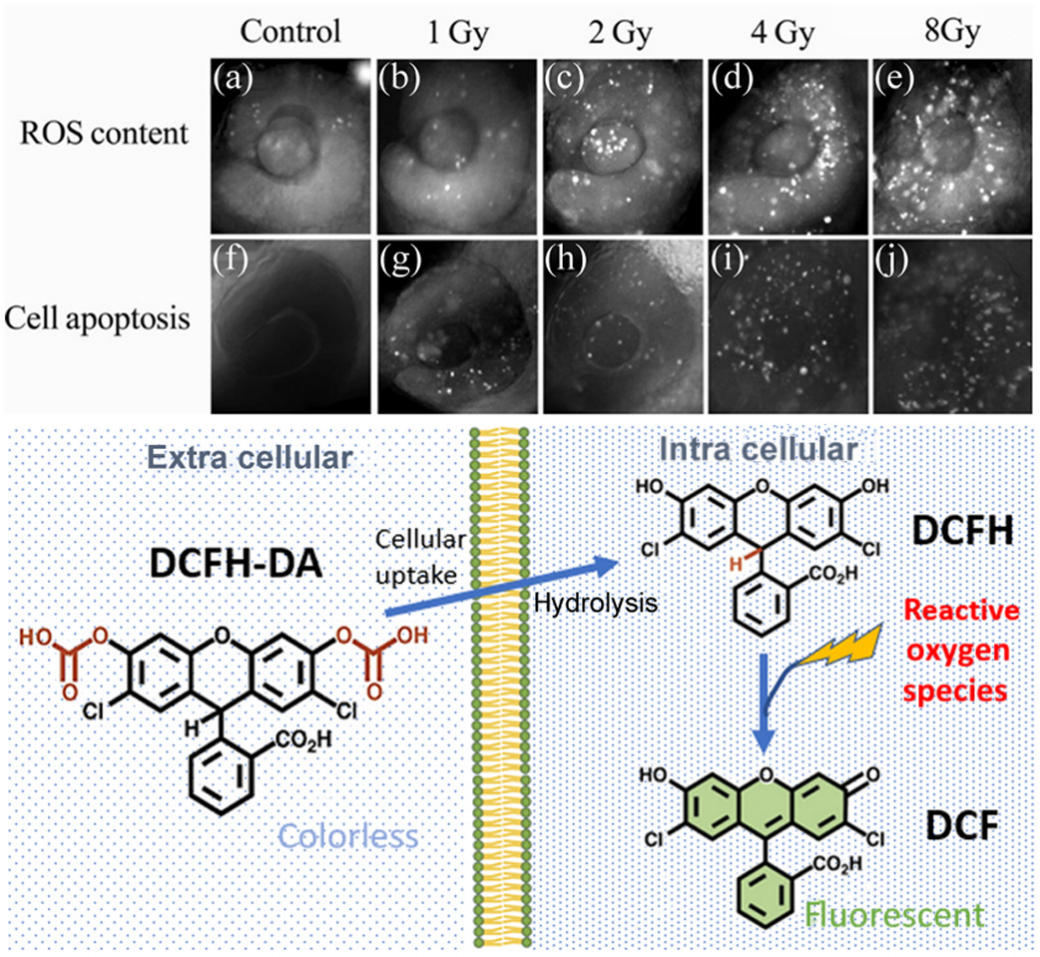
Detection of x-ray radiation-induced changes in ROS content and cell apoptosis in eyes of live zebrafish embryos. (a)–(e) ROS content was measured using the fluorescent dye DCFH-DA. (f)–(j) Cell apoptosis was determined using AO staining. Figure adapted with permission from Ref. 39. The chemical pathway of DCFH-DA from extra cellular administration to hydrolysis with intracellular uptake and reaction with ROS to form the fluorescent DCF molecule is illustrated.^40^

2′,7′-dichlorofluorescin diacetate (DCFH-DA) is a fluorogenic dye that is one of the most common methods for monitoring $H_{2}O_{2}$, as well as a host of other ROS species that are involved. As shown in Fig. 4, DCFH-DA is cell permeable and, upon diffusing into a cell, becomes deacetylated into the form of DCFH, which upon being oxidized by ROS becomes DCF, which is optically active with an excitation peak at 498 nm and emission peak at 522 nm.^41^ Although the assay primarily reacts with $H_{2}O_{2}$, it also reacts with hydroxyls, peroxyl, and peroxynitrite, which shows that it can monitor both ROS and other species.^42^ This assay has been used to monitor *in vivo* mice tumor cells via the use of a tumor window.^43^

CellROX molecule reporters are reported to be primarily reactive with hydroxyl radicals and superoxide.^44^^,^^45^ CellROX is a commercial agent related to DCFH-DA but of unpublished specificity in its molecular structure. It is commonly used in conjunction with other ROS related reporters (BODIPY, MITOSOX Red, and DCFH-DA) to all together assess various ROS related productions in solutions and *in vitro*.^46^^,^^47^ Although cited as detecting primarily superoxide, it is currently a proprietary kit, meaning that only the reported properties by its manufacturer are known and its exact molecular structure is unknown.

### 3.3 Chemiluminescence

**Fig. 5:**
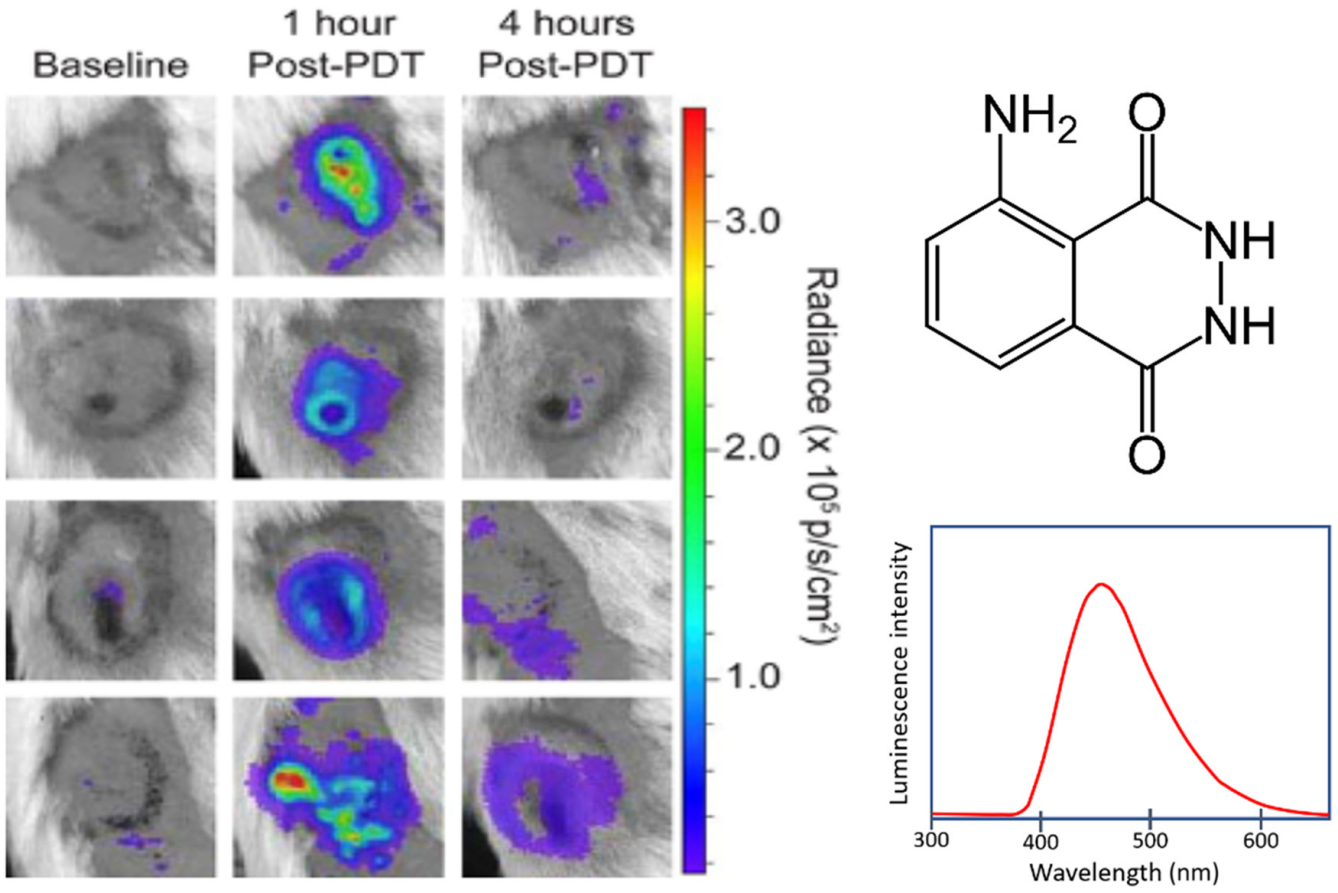
Murine mesothelioma flank tumors (AB12) treated with PDT and imaged for the inflammation response by the chemiluminescent reporter luminol, which reacts with an array of ROS and here shows a notable difference from baseline at both 1 and 4 h post-PDT. Figure adapted with permission from Ref. 48.

Several chemiluminescent reporters of ROS exist, of which luminol is the most widely used. Luminol is a crystalline compound known for its well-characterized chemiluminescence properties, with a UV absorption peak at 355 nm and an emission peak at 425 nm but with broad emission throughout 380 to 600 nm, as shown in Fig. 5.^49^ The large Stoke’s shift makes luminol a useful probe for biological imaging to eliminate self-quenching of the emission; it is excited by black light and has a strong blue emission that provides a larger detectable signal of luminol oxidation. Iron can catalyze the decomposition of $H_{2}O_{2}$, which results in higher luminol oxidation yield, making these molecules commonly used by forensic analysts as an enhanced sensitivity blood detection reagent.^50^^,^^51^

Biochemically luminol binds to human serum albumin. Toxicity data on luminol are limited, and it was used to promote blood clotting and for clinical treatment of alopecia in the 1960s. However, high doses in experimental animals are known to cause pharmacological effects or even death. Due to its potentially mutagenic and toxic nature at high doses, luminol use *in vivo* has become a topic of controversy. It is used in pre-clinical studies at tolerable doses for short-term metabolization and excretion although not for long-term studies.^52^ Recently, *in vivo* use has been investigated methods to for the chemiluminescence measurement of ROS. Nan et al.^53^ developed a micelle that releases a fluorophore for the purpose of imaging $H_{2}O_{2}$ *in vivo* triggered by the chemiluminescence of luminol.

Luminol reacts readily with multiple ROS including $H_{2}O_{2}$ and hydroxide *in vitro* in the presence of peroxidase enzymes.^54^ ROS detection with luminol has been sufficiently characterized and deemed reliable enough to be used as a method for investigating both *in vitro* and *in vivo* radiation damage and how it changes in response to pharmaceuticals and dose variation. Luminol was used alongside lucigenin as a means to determine relative amounts of reactive oxidants ($H_{2}O_{2}$, $O_{2}^{-}$, ${\mathrm{OH}}^{-}$, and HOCl) in the organs of irradiated rodents to assess whether *Ginkgo biloba* extract could protect rats from oxidative organ damage.^55^ Also its use in dose optimization with cell culture was examined to assess limits in irradiation-induced increased phagocytic function, as an indicator of inflammation.^56^

An engineered chemiluminescent probe of both ROS and reactive nitrogen species is a luminol analogue called L-012. L-012 has been used to examine ROS production by UV light on tissue.^57^ This was examined in living mice skin and cells and showed promise toward *in vivo* studies of imagining ROS *in vivo* for disease monitoring. While being similar to both, this was engineered to have both a larger intensity at its peak of ∼400  nm and improved specificity. One primary issue with L-012 is that it primarily reacts with superoxide, but this interaction needs to be facilitated via the presence of a peroxidase.^58^

Lucigenin is another chemiluminescent molecule with an excitation/emission peak at 369/503  nm; it is similar to luminol but is thought to be more specific to superoxide rather than luminol’s primary sensitivity to a mixed response to $H_{2}O_{2}$, superoxide, and hydroxide. Reports of lucigenin undergoing redox cycling have created apprehension about the utility of lucigenin in complex biological systems.^59^

### 3.4 Photobleaching

Photobleaching is a less common assay of ROS damage, but it is well known to exist and be quantified in many assays. The strength of this approach is that it can be measured *in vivo* or even intracellularly via microscopy. The use of this as a tool to assay oxidative stress has been reported, attributing the majority of the bleaching to $H_{2}O_{2}$.^60^ Modifications to fluorescein can be achieved to protect the molecule and make it more localization specific or sensitive to certain species, although this is not widely available.^61^ However, although this assay can be simple, careful interpretation must be done because intracellular localization site and biological changes, such as loss of membrane polarization, can significantly shift the fluorescence.^62^ Still, in terms of an *in vivo* assay that can be utilized to compare radiation effects between tumor and normal tissue, this could be one of the more translatable ideas.

**Fig. 6:**
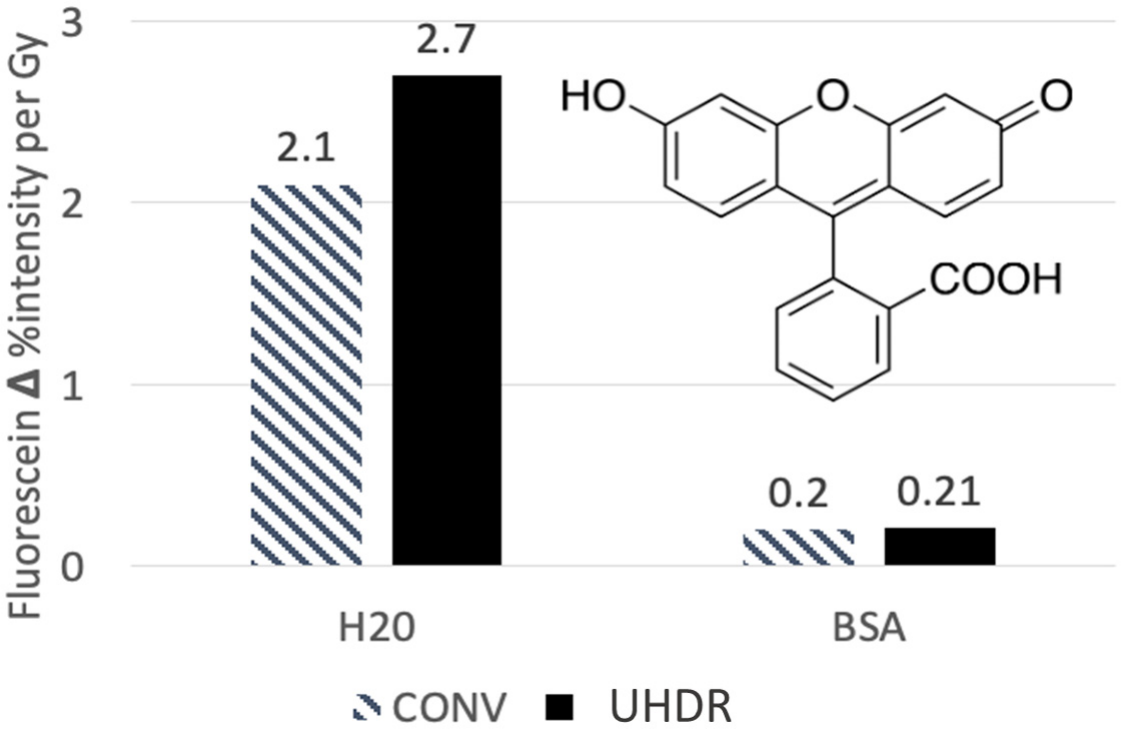
Photobleaching of 2  μM fluorescein measured in water and 5% bovine serum albumin solution, using conventional dose rate (0.03  Gy/s, denoted CONV) and UHDRs (100  Gy/s, denoted UHDR), illustrating the change in mechanisms with dose rate. The fluorescence intensity loss was measured as % decay in signal per Gy delivered to the solution.

This was used by our group in recent studies for characterizing the radiochemical differences of dose rates in both water and protein assays. In this case, the response of fluorescein in water solutions was quite substantial, corresponding to a 2% to 3% signal decrease per Gy delivered, as shown in Fig. 6. When translated to a protein environment with concentrations 100 times that of the fluorescein, the signal change was minimized by a factor of 10, showing that, when proteins are involved, the fluorescein photobleached substantially less, resulting in less application of the assay toward future in vivo tests.

### 3.5 Bioluminescence

The use of bioluminescent properties via luciferase for optical reporting is widespread. This methodology is the same functionality as what enables fireflies to light up. The distinction from fluorescence is that the light production comes from the breaking of a chemical bond rather than an incident photon. The engineered aspect of this reporter is chemically modifying both the luciferin substrate and the luciferase enzyme to produce red shifted lights for better tissue penetration. One aspect of the engineered reactions that results in a red shifted signal is changing the charge distributions on the resulting produced oxyluciferin.

**Fig. 7:**
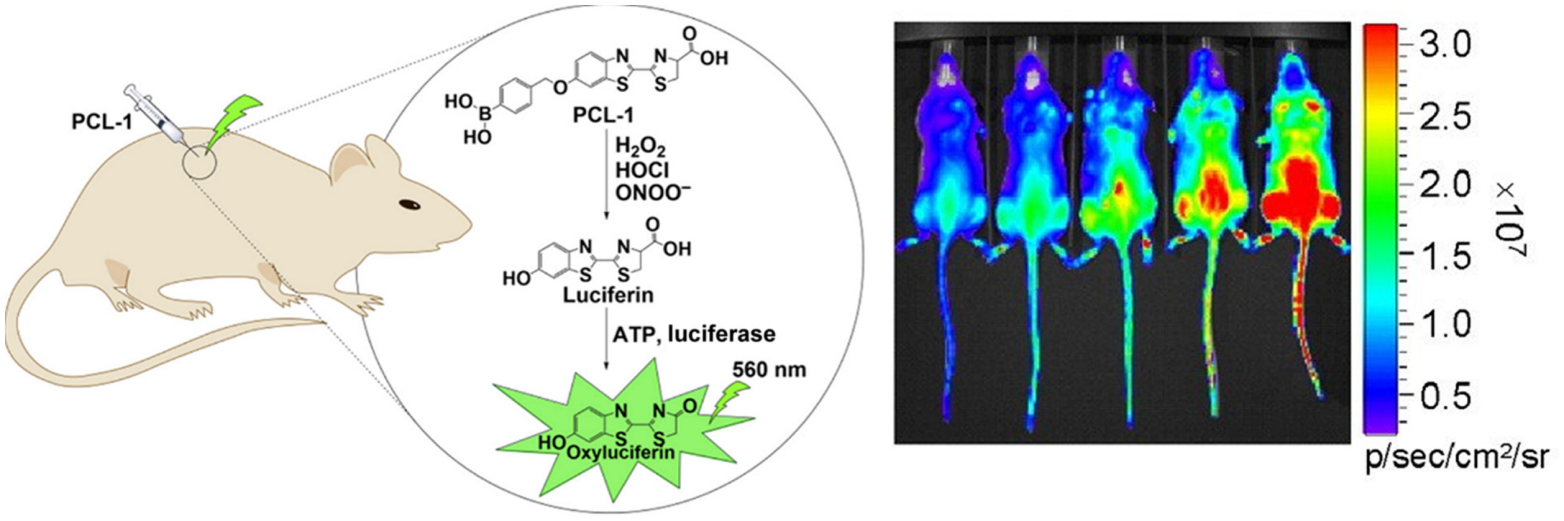
Conceptual framework of caged luciferin (PCL-1) that is released by reaction with $H_{2}O_{2}$, HOCl, or ${\mathrm{ONOO}}^{-}$ for bioluminescent detection of ROS *in vivo*.^58^ *In vivo* images show the 30 min post-injection image of mice injected with PCL-1, which upon interacting with $H_{2}O_{2}$ show emission. The amount of $H_{2}O_{2}$ injected scales from 0 to 24 mM going from left to right.^63^

As the system functions purely via luciferase and luciferin, a functionality must be implemented to detect the ROS; these are called caged bioluminescent probes. The primary example of a chemically engineered bioluminescent system for ROS detection *in vivo* is peroxy caged luciferin (PCL-1), which has a 614 nm emission.^58^^,^^63^ PCL-1 functions via being administered into the system with a luciferase enzyme and luciferin substrate. Once in the system, the peroxide cage is oxidized selectively by hydrogen-peroxide and to a lesser extenthypochlorite or peroxynitrite; it then turns into D-luciferin, which is finally oxidized by the injected luciferase to produce the detected light signal. Its application in mice can be seen in Fig. 7.

## 4 Lipid Peroxidation Reporters

A few optical reporters of biomolecular damage that can be used in cells have been developed,^64^ but their specificity lies largely in their partitioning for the molecules of interest. Specificity to exact ROS species is rare, but partitioning into cellular components allows an assay to at least be specific to the region of the cell where it is present. One approach widely examined is lipid peroxidation assays, which are determined by the partition coefficient and by careful choice of targeting moiety. The emerging field of ferroptosis has spawned considerable interest in better quantifying lipid peroxidation using fluorescent reporters.^65^ Through binding to the site of interest within a heterogeneous mixture, it can report on peroxyl formation instead of just ROS in solution.^66^ The molecule 4, 4-difluoro-5-(4-phenyl-1,3-butadienyl)-4-bora-3a,4a-diaza-s-indacene-3-undecanoic acid, termed BODIPY, is widely used as a ratiometric fluorescent ROS sensor, but various versions of the molecule have been created for localization to specific sites, which allows it to have more specificity to different molecular damage sites. The native version of BIODIPY 665/676 is a lipid peroxidation sensor because it is highly lipophilic, so it localizes at the site of the peroxidation molecules.^67^ However, testing of the localization and verification that the reporter change is from lipid peroxidation versus just the ROS molecules themselves is challenging.

One translation of BODIPY 581/591 undecanoic acid to intracellular sensing was done recently in a new reporter termed MitoCLox,^68^^,^^69^ which was designed to measure peroxidation of the inner mitochondrial membrane. It is composed of C11-BODIPY (581/591) probe conjugated to triphenylphosphonium cation via a flexible linker with amide bonds, and its oxidation is observed as increased fluorescence at 520 nm and decreased at 590 nm wavelengths. MitoCLox is a positively charged molecule that binds to the negative cardiolipin in the inner mitochondrial membrane. The peroxidation of cardiolipin is thought to be a highly sensitivity site for ROS, and this is involved in the initiation of apoptosis. Thus MitoCLox might be used to observe peroxidation cardiolipin in living cells.

## 5 Oxygen Reporters

Oxygen measurement in tissue has had a long history,^70^ matching the studies of radiobiology effects, largely because of concerns about chronic or transient hypoxia reducing the radiation damage to tumors, from the reduced oxygen enhancement factor.^71^ However, perhaps most importantly, a number of studies have shown that the loss of oxygen due to radiochemistry *in vivo* can be measured when very high radiation dose rates are used.^72^^,^^73^ The reaction dynamics of oxygen in tissue had not been considered much before this, beyond the theoretical belief that peroxyl radicals were involved in DNA damage fixation.^74^ The reactions of oxygen are well known to be involved in DNA damage, and the biological manifestation of this is through the well know oxygen enhancement ratio (OER).^75^^,^^76^ Since discovery of the biological tissue sparking from UHDR FLASH in 2014, the amount of the depletion of oxygen has been widely studied. However, now it appears that there is not depletion to the point of radiobiological hypoxia. Still, a key feature of UHDR irradiation is that there is actually a reduction in the magnitude of oxygen consumption with increasing dose rates, and this can be quantified with careful measurement of the fast transients.^72^^,^^73^ This observation may be a surrogate for damage, under the assumption that oxygen consumption is what leads to peroxyl radical formation, and reductions in this lead to less damage.^77^^,^^78^

**Fig. 8:**
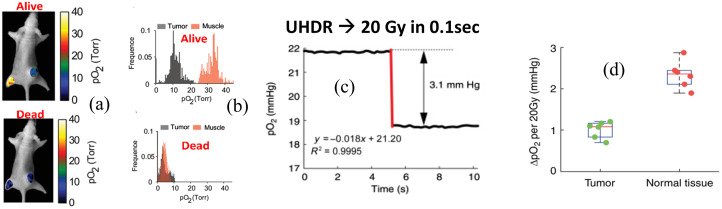
(a) Oxygen measurements can be acquired *in vivo* with Oxyphor phosphorescence lifetime measurement and (b) imaged with phosphorescence lifetime imaging to show histograms of ${\mathrm{pO}}_{2}$ when excited by the radiation beam. (c) In separate experiments, the transient changes in ${\mathrm{pO}}_{2}$ were quantified during UHDR radiation treatment to quantify the change in oxygen, due to radiation chemistry-based consumption. (d) Differences in oxygen from UHDR irradiation of 20 Gy in 0.1 s could be seen as an abrupt decrease in both normal tissue and tumor tissues.^72^

The measurement of oxygen has always been challenging, with electrodes tending to be the standard tool for absolute data,^79^^,^^80^ but more recently there has been a number of phosphorescent quenching probes that work *in vivo* and have been applied to measure oxygen dynamics.^81^^–^^83^ Molecular oxygen is one of few abundant species that exists as a triplet electron spin in its ground state, which makes it possible to receive collisional energy transfer readily from other excited triplet state molecules. Several metalloporphyrins have excited triplet states that are near resonant with oxygen, allowing for photodynamic production of singlet oxygen this way.^84^^,^^85^ The diffusion of oxygen molecules provides the process for quenching these triplet states. For phosphorescent dyes dissolved in tissue and excited by a pulsed light source, the triplet decay or phosphorescence lifetime becomes a quantitative reporter of the local oxygen concentration. The loss of phosphorescence and the apparent lifetime change is a direct indicator of the degree of collisional quenching from oxygen and hence is an indirect way to quantify the oxygen present in its immediate environment. Several investigators have used this methodology to quantify and image oxygen *in vivo* using metalloporphyrins.^86^^–^^89^ This is illustrated in Fig. 8.

**Fig. 9:**
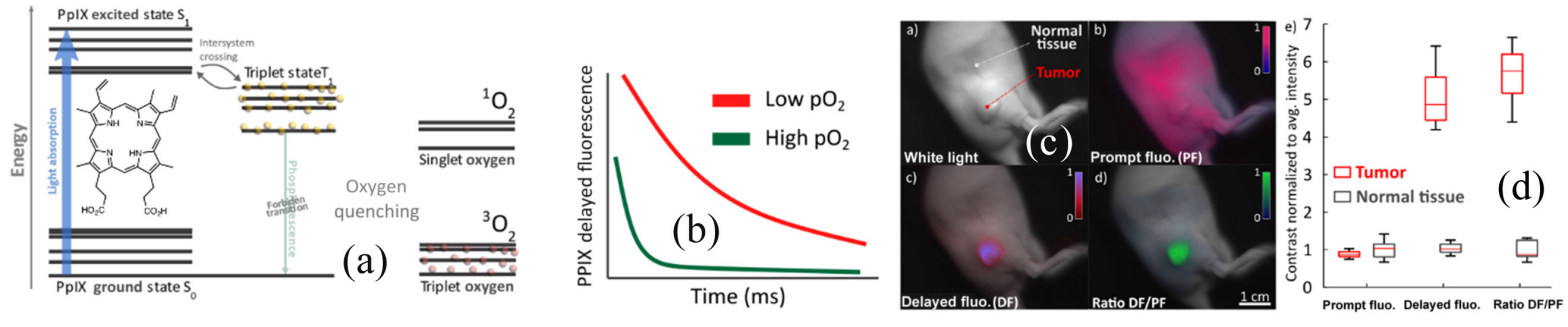
Oxygen measurements can be achieved *in vivo* through DF from protopoprphyrin IX molecules generated within cells. (a) This compound has a triplet state quenched by oxygen, and when not present, there is considerable reverse intersystem crossing to allow for (b) increased DF signal. In mice, (c) this DF can be seen from hypoxic tumors, and (d) the contrast relative to normal skin is high compared with the prompt fluorescence. The ability to measure oxygen with this molecule is evolving now.^95^

In comparison with exogenous agents, there is one documented endogenous agent that provides an optical signal of tissue oxygen *in vivo*, namely protoporphyrin IX.^90^^–^^92^ It has been shown that this molecule, as well as a few other exogenous porphyrins,^93^^,^^94^ has reverse intersystem crossing from states triplet to excited singlet states, which produces a delayed fluorescence (DF) with a lifetime that is dictated by the triplet state. The measurement of the DF lifetime is a direct measure of the collisional quenching of the triplet state by molecular oxygen, so the resulting lifetime and emission intensity can be interpreted as related to the local oxygenation. Because protoporphyrin IX is produced intracellularly at the mitochondria, it is a signal that is predominantly directly from the cells themselves versus the vascular or interstitial spaces. This has been used to visualize oxygenation in skin and tumors^95^ and is illustrated in Fig. 9.

The ability to measure oxygen decreases from radiation is challenging, given that the vascular supply provides a continuous reperfusion and diffusion of oxygen. However, if the radiation delivery is a fast sub-second pulse, such as in UHDRs, then it is likely possible to directly measure the loss of oxygen *in vivo*, as documented recently.^72^ The change in oxygen can be related to lipid peroxidation, given that this is one of the more dominant pathways of damage during the time scale of the radiation pulse and in the microseconds after it.

## 6 Linkage to Biological Damage Assays

**Fig. 10:**
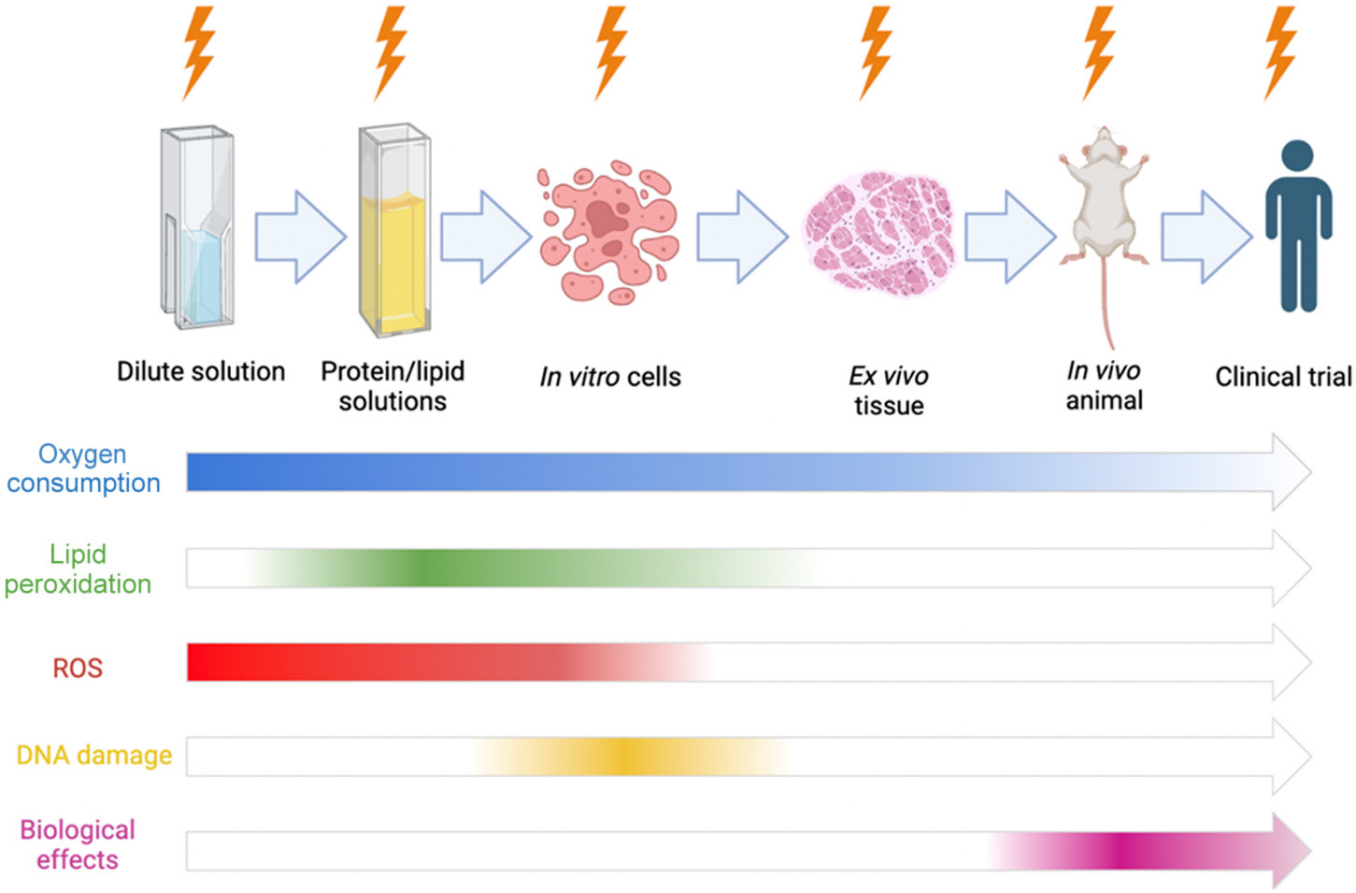
Schematic illustrating the available reporters for *in vivo* radiation effects that could be used, for each step-in scaling from irradiation of protein solution to *in vitro* cells to *in vivo* experiments with eventual translations to clinical trials. Most optical reporters cannot translate to human use, but a few are emerging to translate to *in vivo* experimental use.

This survey of optical reporters of radiation damage and effects was undertaken with the purpose of identifying, highlighting, and hypothesizing which would have the most value for testing new advances in radiotherapy technique. The limitations of most biological assays are well known, with the primary one being heterogeneity of response, which limits the ability to discern small changes. This is illustrated in Fig. 1(e), which shows that, for example, if the biological assay has 30% standard deviation, then it becomes nearly impossible to discern if an improvement in a radiotherapy delivery technique has a 10% benefit. However, a 10% benefit in technique could have a large benefit if applied in clinical practice. So the tools existing to assay new therapies have clear limits. Although radiochemical assays would never fully replace biological response as an endpoint assay, they can provide surrogate information that can help reduce the number of biological endpoint assays that are required in a complex set of choices for radiotherapy, as illustrated in Fig. 10. Second, these assays can provide clues to the mechanisms of action that underlie subtle changes in radiotherapy efficacy that might not be clearly understood.

**Fig. 11:**
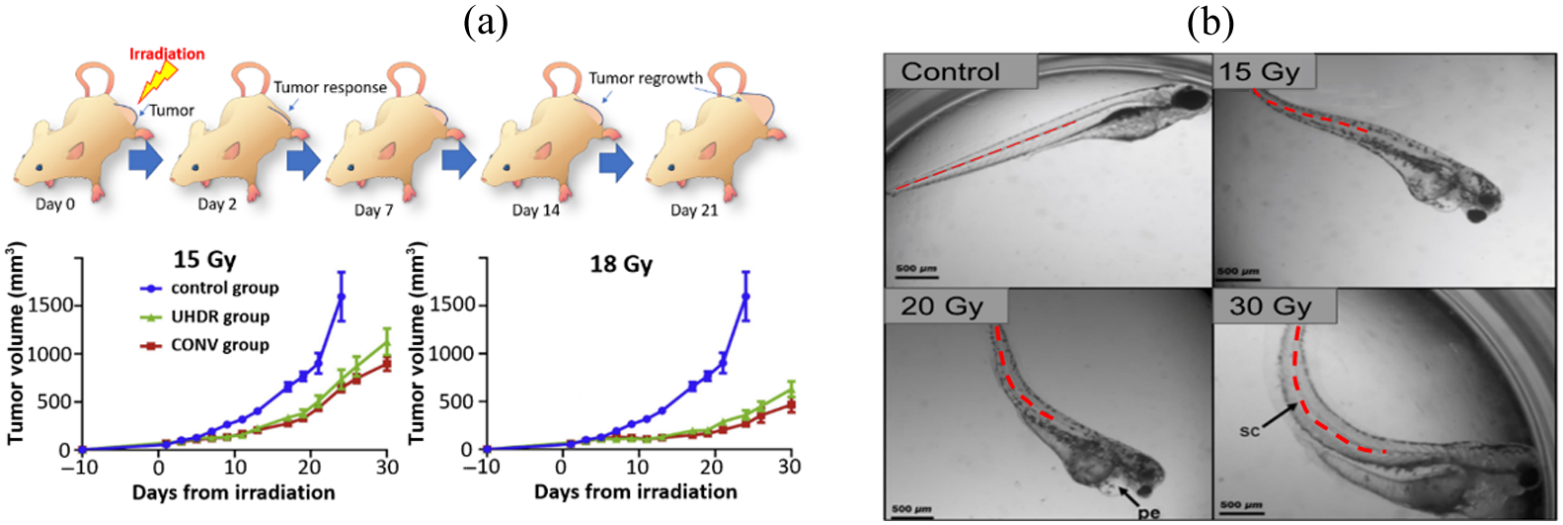
Visual illustrations of response measures. (a) Tumor regrowth assay from tumor volumes measured.^99^ (b) The zebrafish embryo undergoes the acute, quantifiable biological effect malformations observable from spine curvature.^100^

The need for reporters of damage that can be more directly linked to the initial radiation dose deposition event, or first site of radiation damage, is most compelling when compared with the imprecision of biological assays *in vivo*. The most widely used visual change in radiation damage has been tumor volume change as this is the goal of most radiation therapy, and the visual measurement of tumor size is seen as the best indicator of efficacy.^96^ However, the variability of this type of assay is quite high,^97^ and the reliability is compromised by variations in tumor line passage, cell subpopulation variation,^98^ tumor size at the start of irradiation, tumor necrosis variation, and even simply animal temperature and health. As such, although this is perhaps the *de facto* standard for comparing efficacy prior to large animal or human studies, it has inherent high variability and potential for unintentional user bias. An example of this assay is illustrated in Fig. 11(a),^99^ in which FLASH radiotherapy was delivered to pancreatic tumors in mice, but the subtle differences in response between conventional (CONV) and UHDR radiotherapy responses were not visible for two different dose levels. The variation in response between animals combined with subtle changes in the average response can lead to this assay not providing useful information.

A recently adopted assay for evaluating radiobiological damage is by measuring the effects in the zebrafish embryo.^39^^,^^101^^,^^102^ Zebrafish are unique in that their flesh is transparent, which allows for optical imaging of changes in anatomy as a response to radiation.^103^ One visually apparent anatomical alteration induced by radiation injury is a curling of the spine as the embryos grow, as seen in Fig. 11(b).^100^ Notably, Montay-Gruel et al.^103^ studied how spine length and shape change as a result of the delivered dose as a part of a larger investigation on comparing late effects from conventional versus UHDRs. They determined the the primary driving factor in the observed deformations was oxidative stress resulting from ROS interactions.

**Fig. 12:**
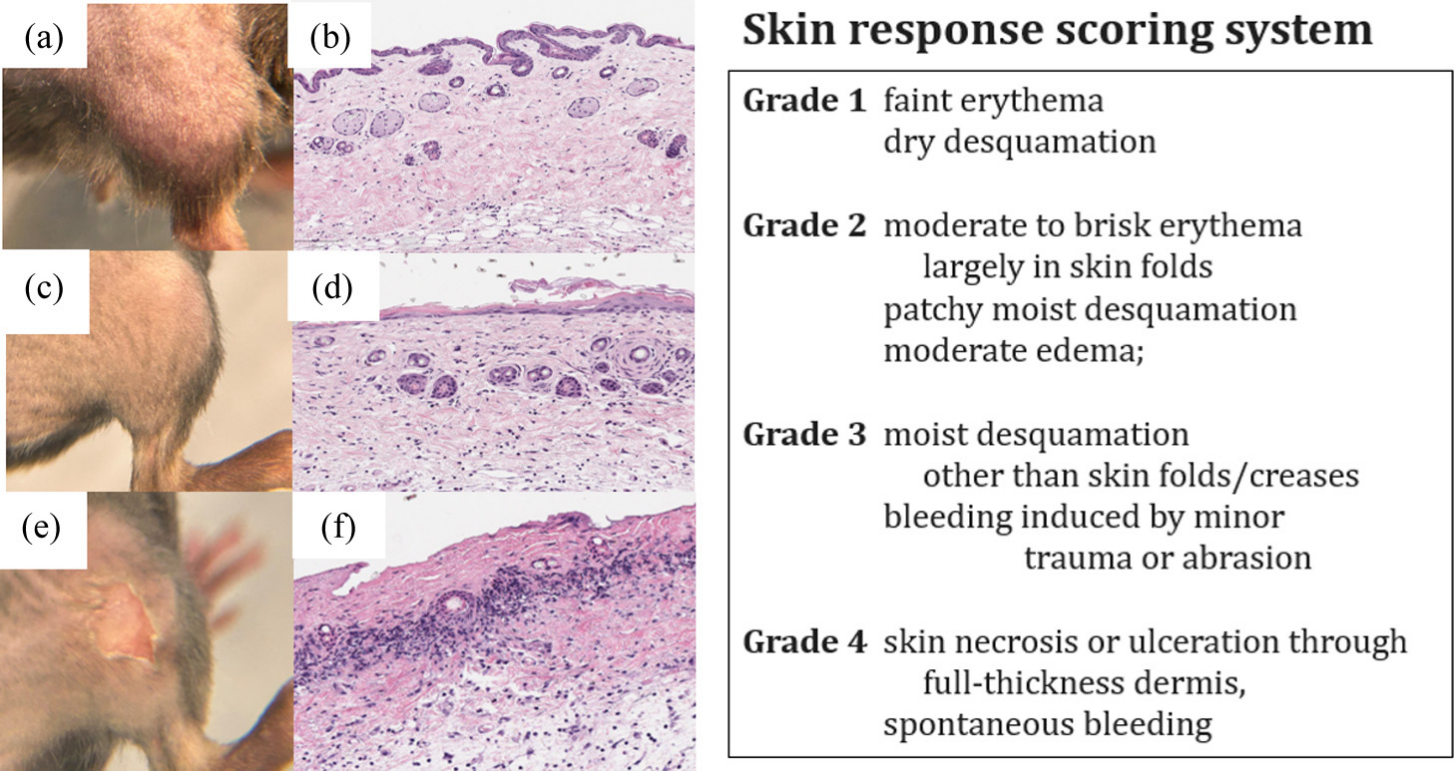
Illustration of skin damage occurring in mice skin going from: (a) clinical appearance of shaved normal mouse skin; (b) histological appearance of normal mouse skin with 3–4 cell thick epidermis, normal hair follicles and sebaceous glands in dermis; (c) clinical epidermal desquamation in irradiated shaved skin; (d) histology of irradiated skin with loss of epithelial viability, sebaceous gland loss and excessive fibrosis; (e) clinical classic radiation induced skin most desquamation and ulceration; and (f) histologic radiation induced full thickness epidermal ulceration and necrosis, loss of epidermis and adnexal structures, with marked dermal inflammation and fibrosis.^104^ At right, a narrative illustration of one skin scoring system utilized for radiation response, often in combination with other clinical factors.^105^

Common side effects from radiation are radiation melanosis or radiation dermatitis (RD), resulting from the cutaneous radiation injury. CDC recommendations grade these from I to IV based on the amount and type of observable symptoms, although there is a lack of standardization in research. One stated scoring system is listed in Fig. 12.^105^ Within this area, one issue is that multiple terms are often used interchangeably to describe similar skin conditions, such as cutaneous radiation injury, local radiation injury, RD, radiation-induced skin injuries, burns caused by radiation oncology procedures, and radiation burns.^106^ Differences in response are known to have a dose response, although the biological heterogeneity of these responses does make it challenging to use them as a metric of dose, and of course mouse skin response does not translate quantitatively to humans. RD has been used to study patients receiving protons versus photon therapy, for example,^107^ to quantitatively compare their value in skin sparing.^108^ However, the dosimetric uncertainty at the skin combined with the high variability between subjects leads to concerns of the inability to draw conclusions about small dose differences’ contributions to average skin toxicity. Commonly optical luminescent dosimeters or thermoluminscent dosimeters (TLDs) are used in surface dosimetry of patients with an accuracy of ±5%, with steep dose gradients and major problems with scatter contributions limiting biological assays that are superficial. Patients receiving whole chest wall radiotherapy are known to potentially experience decreased levels of skin hydration, sebum content, erythema, and melanin levels as symptoms of their radiation exposure.^107^ Each of these can be permanent, except erythema as RD improves over time, but melanin levels may not return to baseline levels, even after 3 months of therapy completion.^109^

Perhaps no other area of radiotherapy research is as intensely studied today as the role of immune infiltration and immune reactions, either as mechanisms to synergize response or to understand how to amplify tumor damage.^110^ The assessment of ROS is highly relevant to the immune response^111^ because it is thought to be a driving force in initiating immune infiltration as well as likely altering local responses. Thus the ability to use *in vivo* optical reporters has substantial value in improving radiobiological impacts of immune response and immunotherapy. Radiotherapy activates both the innate and the adaptive immune responses through the production of radicals in tissue,^110^ linking optical assays to immunological assays could result in further understanding of downstream effects and thus further optimization of RT.

## 7 Discussion

The central hypothesis of this work is that biological variability is extremely high in downstream effects from radiation exposure, as illustrated in Fig. 1, such that response curves can be overwhelmed by the magnitude of the uncertainty. This is problematic for small changes in treatment techniques that might only have a small percentage improvement in response but where the variability of the assay makes it impossible to objectively determine the benefit of the technique change. It would always be more desirable to have quantitative assays that can detect subtle changes in damage at a level that minimizes animal use and provides objective conclusions. It could be argued that a large fraction of radiobiology has been confounded by the issue that the variability in response among animals simply overwhelms the ability to make useful conclusions. Although radiochemical measurements are not a complete replacement for biological damage assays, they provide some level of surrogate measurement that can complement the biological assay. Ideally, as illustrated in Fig. 10, the radiochemical assays might have translation potential to go beyond solution work to *in vitro* cells or *in vivo* animal tissues. If <20% are needed to augment biological assays, it reduces the number of animals needed for assessment and makes the choices more informed. The areas of application are multiple and varied in radiotherapy research and development. The primary ones today that are highly studied are in the following fields:

(1) radiation sparing treatments for certain organs based on technique, design, or procedures
(2) radiation sensitizers to amplify locally deposited damage
(3) radiotherapy-immunology interactions (ROS stimulation)
(4) radiation source choices (photons, electrons, protons, and heavy ions)
(5) spatially fractionated radiation therapy (GRID and microbeams)
(6) UHDR (FLASH) radiotherapy.

The advancement of each of these techniques has the same goal, which is to maximize the tumor damage while minimizing the long-term damage to normal tissue or organs at risk. Successes in this space can be small, and even 5% to 10% benefits in this goal can have significant clinical importance. Establishing more efficient and accurate assays of *in vivo* damage can have benefits of providing convincing evidence that one technique appears superior to another technique and that pre-clinical data might provide a way to minimize the use of excessive animals in subsequent research, as well as giving statistical information about how to power a study. Many studies in radiobiology have led to confounding or unclear results because of the limitations of simple biological assays, such as tumor regrowth or DSBs, or *in vitro* work that did not lead to similar results *in vivo*. The assays discussed here were chosen as ones that might point to better clarity in results when the net therapeutic ratio benefit might be marginal.

When investigating the underlying radiochemistry of water radiolysis, there are variety of assays that are applicable as a cell is not simply a vessel of water, and translating water-based results toward *in vivo* interpretation is key in making conclusions of radiochemistry in normal tissue for the assessment of changes that would impact the therapeutic ratio. This is the general barrier to using these quantitative assays. As it stands, there are thousands of assays that can detect ROS *in vitro*^64^^,^^112^^,^^113^ as these are tested for potential use *in vivo*, and the relevant stable assays are radically fewer, as shown schematically in Fig. 10 and tabulated in Table 1. By going through each series of assays in environments scaling from water to protein solutions to *in vivo* usage, it is possible to create a workflow for examining variations of radiation therapy modalities that systematically shows how they relate to biological outcomes.

**Table 1:** Tabulation of optical reporters. They are thought to report on ROS in different ways, listed by their experimental application, spectral properties, site of localization, known molecular sensitivities, and if they require catalysts.

Optical reporter	Primary ROS activity	In solution	In cells	*In vivo*	Ex /Em (nm)	Site of localization	Molecular sensitivity	Catalyst
Fluorescent ROS reporters								
Amplex Red	$H_{2}O_{2}$	√	√		570/585	Cellular		N/A
CellROX Red	OH•, ${O_{2}}^{-}$	√	√		644/665	Cytoplasm		N/A
DCFH-DA	$H_{2}O_{2}$	√	√	√	498/522	Cellular	OH, ROO•, ${\mathrm{ONOO}}^{-}$	N/A
Chemiluminescent ROS/NOS reporters								
Luminol	$H_{2}O_{2}$, ${\mathrm{OH}}^{-}$	√	√	√	355/425	HSA	ROO•, ${\mathrm{ONOO}}^{-}$, $O_{2}^{-}$	Peroxidase
L-012	$O_{2}^{-}$	√	√		—/400	HSA	NOS	Peroxidase
Lucigenin	$O_{2}^{-}$	√	√	√	369/503	HSA		Peroxidase
Photobleaching fluorescent ROS reporters								
Fluorescein	$H_{2}O_{2}$	√	√		498/517	Varying sites	OH, ROO•, ${\mathrm{ONOO}}^{-}$	N/A
Bioluminescent reporter								
PCL-1	$H_{2}O_{2}$	√	√	√	—/614		${\mathrm{ONOO}}^{-}$	Luciferase
Lipid peroxidation fluorescent reporters								
BODIPY 665/676	Peroxyl radicals	√	√		665/676	Peroxidation molecules		N/A
MitoCLox	Peroxyl radicals	√	√		581/591	Inner mitochondrial membrane		N/A
Phosphorescent oxygen reporters								
Oxyphor G4	Molecular $O_{2}$	√		√	698/813	Extracellular	$\Delta {\mathrm{pO}}_{2}$	N/A
Protoporphyrin IX	Molecular $O_{2}$	√		√	405/635	Mitochondrial, extracellular	$\Delta {\mathrm{pO}}_{2}$	N/A
Oxyphor R4	Molecular $O_{2}$	√		√	428/448	Extracellular	$\Delta {\mathrm{pO}}_{2}$	N/A
Ruthenium complexes	Molecular $O_{2}$	√	√	√	470/580	Cytoplasm, membrane	$\Delta {\mathrm{pO}}_{2}$	N/A

Finding an optical probe to show the presence of a calibrated and proven specific ROS species (i.e., $H_{2}O_{2}$ or superoxide) has been a focus of many studies; however, utilizing any of these probes to show specificity of reactivity to one ROS molecule in the presence of multiple ROS species has been nearly impossible. Generally, when creating optical probes, a single-assay assessment is done for probe evaluation, and when evaluating radiolysis, there is a variety of ROS being injected into the system simultaneously. This means that one may need to evaluate whether an assay for one ROS is impacted by another ROS to ensure accurate conclusions. Furthermore, proving that there is a 1:1 reaction between the probe and the radical is challenging in a dilute solution,^112^ and extrapolation of this to a heterogenous medium of proteins or *in vitro* cells is not easily proven. A key factor in using optical reporters at low concentration is their need to physically interact with the target ROS molecule to provide a detectable effect through reaction. These temporal reaction-kinetics depend on the rate constants of the reaction and the concentrations of the species involved. The reaction rates depend on free diffusion, and the reaction rate is a function of the solubility and viscosity in the medium. In complex systems, if the target molecule cannot diffuse into the optical reporter, a detectable measurable effect will not be seen. An example of this is longer-lived radical species, such as $H_{2}O_{2}$, which can diffuse and react with optical reporters on long time scales. In contrast, hydroxyl radicals are highly reactive and have a lifetime near 10 ns, so they react too quickly and locally, thus resulting in minimal interaction with low concentrations of optical reporters. The use of complex, biologically relevant systems further slows down the diffusion, altering and reducing the potential to observe this species.^64^ Optical reporters of ROS are key for developing insight into the fundamental radiochemical differences of varying radiation delivery modalities via examining the observable ROS that directly causes damage to DNA. Radiation induced DNA damage is broad in scope with multiple pathways that cause damage without a clear indicator of the amount of damage from each individual path and how that quantitatively relates to macroscopic biological impact.^114^ The quantitative aspect of these reporters for observing radiochemical effects of radiation therapy is key in developing conclusions of underlying radiobiology.

An area of high recent research interest in ROS is lipid/protein peroxidation.^64^ The kinetics of lipid peroxidation have been specifically examined because of how complex the chains of subsequent ROS generation can be.^68^^,^^69^ Although the most widely used commercial lipid peroxidation reported is based on BODIPY, studies to date have been restricted to *in vitro* membranes or *in vitro* cell work. The use of this reporter has not been extended to *in vivo* measurement, and it is not clear that it would be possible. Although currently not at the point of use for *in vivo* experiments, use of protein assays and cells as a model.^115^ Developing an understanding of how varying radiation modalities impacts lipid peroxidation is a key aspect in developing fundamental hypotheses on radiation response.^116^

Although oxygen is not a direct source of damage, its role in producing ROS is the key factor in most secondary damage within cells and tissue. The presence of oxygen is characterized in radiobiology by the OER. The OER is an empirical parameter that provides information about tissue to tissue variability in oxygen enhancement of damage, but it provides no mechanistic insight into the cause of this. The core hypothesis has been that oxygen “fixes” damaged DNA through peroxyl formation of the broken nucleotides.^74^^,^^117^ This fixation has not been explicitly proven, but the concept of creating more permanent damage in DNA from the involvement of oxygen or ROS is widely accepted. The most striking thing about oxygen is that it can be quantified in all forms of assay, from dilute solution to cells to *in vivo* and into human clinical trials (see Fig. 10). The second key factor is that radiation consumption of oxygen can be observed and quantified to high precision, even *in vivo* (see Fig. 8). Thus measurement and monitoring of acute oxygen changes during radiotherapy, especially UHDR FLASH treatment, can provide an assay of damage effects that may be related to oxygen fixation and the OER effect. The measurement of oxygen and the change in it may be one of the only reliable means to sample radiochemistry *in vivo* across all models and tissues.

The above reporters must be linked to assays of DNA damage to have relevance as these are the standard for observing radiation damage *in vitro* and are widely believe to report on the key effect or radiation damage.^12^^,^^118^ Due to the cellular preparation steps and complexity of each, the translation of DNA damage assays to *in vivo* use is very limited. The closest would be studies that utilize *in vivo* irradiation followed by *ex vivo* testing,^119^ but these tend to be focused on blood or other easily separated cells.^120^ Unfortunately, reporters of DNA damage generally also have substantial variation, mainly because the biological response to damage has high heterogeneity.^12^^,^^119^ Still, linking ROS reporters to DNA damage will require mechanistic studies that systematically develop these assays in parallel under controlled conditions, such as the pathway outlined in Fig. 10.

Acute biological effects are also key possibilities for linking to optical ROS reporters. However, as noted above, most acute effects of biological response to radiation in animals or humans have extremely large variability because of many other physiological factors that vary response.^121^ However, the use of optical reporters that can be used in humans, such as Acridine orange, fluorescein, and protoporphyrin IX, are perhaps the best opportunity to allow for *in vivo* ROS sampling in the same living systems as acute response monitoring. Systematic studies of these comparison assays should be carried out to determine if it is possible to mechanistically link ROS to acute biological effects.

## 8 Conclusions

There are a variety of optical reporters or ROS and radiation damage that can be used to generate quantitative measurements of key radiochemical interactions with high precision of their effects. The opportunity to utilize these for improvements in radiotherapy techniques is of paramount importance as they can provide preliminary data that reduces the over reliance on animal studies and provide mechanistic information about the biology. The key steps in making this useful are finding those assays that allow for translation from solution work to *in vitro* cellular work and further the translation to *ex vivo* or *in vivo* experimental work. Several important fluorescent and bioluminescent assays exist though that provide this translation potential. Still, there are very few assays that allow for ROS measurement *in vivo* into human clinical trials, but oxygen measurement is one of these. Further exploration of acute oxygen measurements in UHDR FLASH radiotherapy is a key opportunity to identify irradiation methods that maximize oxygen fixation and oxygen enhancement ratio effects. The linkage between these ROS measurements and the biological assays must be completed to ensure that ROS data are linked mechanistically to biological effects, either in acute assay or late term effects.
